# COVID‐19 and post‐traumatic stress disorder: A vicious circle involving immunosuppression

**DOI:** 10.1111/cns.13431

**Published:** 2020-07-17

**Authors:** Xiao Liang, Yuncheng Zhu, Yiru Fang

**Affiliations:** ^1^ Department of Anesthesiology Affiliated Wuxi Clinical College of Nantong University Wuxi China; ^2^ Clinical Research Center & Division of Mood Disorders Shanghai Mental Health Center Shanghai Jiao Tong University School of Medicine Shanghai China; ^3^ CAS Center for Excellence in Brain Science and Intelligence Technology Shanghai China; ^4^ Shanghai Key Laboratory of Psychotic disorders Shanghai China

**Keywords:** COVID‐19, immunosuppression, post‐traumatic stress disorder, SARS‐CoV‐2

Dear editor,

Since the middle of December 2019, human‐to‐human transmission of coronavirus disease (COVID‐19) has occurred among close contacts.^1^ It has been confirmed that 7 199 313 infections and 408 732 deaths worldwide with a death rate of 5.68% (up to June 9 according to real‐time big‐data reports). Asia, Europe, and America are becoming the most affected pandemic outbreak areas. Until now, global attention has largely been focused on infected patients with Severe Acute Respiratory Syndrome Coronavirus 2 (SARS‐CoV‐2) and physical and psychological states of the frontline medical workers in recent society. With the rapid development of information technology, psychological influences spread more widely via the “We Media,” which is a new media tool beyond what existed at the time of the SARS outbreak in 2003.^1^ Therefore, COVID‐19 represents a psychological challenge, both for those who experience it and healthcare providers. From observations surrounding the SARS outbreak, such challenges are likely to lead to a secondary disaster due to stress and psychological distress,^2^ even after the COVID‐19 outbreak is over. Severe psychological stress factors are highly likely to induce serious mental illnesses and promote post‐traumatic stress disorder (PTSD) in particular.

## 1 IMMUNOSUPPRESSION OF PTSD

PTSD is defined as a stress‐related disorder with subsequent autoimmune disease that may arise after exposure to a serious traumatic event or injury.^3^ It is suggested that PTSD conforms with a bi‐phasic stress response model: Acute stress may reflect an enhancement of the immune response while chronic stress may reflect a suppression of the immune response with increased susceptibility to infections. Therefore, these correlations pose a complex question regarding the conversion from T‐helper 1 cells (Th1) to T‐helper 2 cells (Th2).

A study showed that chronic stress elicits the simultaneous suppression and enhancement of the immune response via alteration of the cytokine expression pattern.^4^ In the chronic stress model, CD4^+^ Th1 subsets release Th1 cytokines that activate the inflammatory cellular immune response. The response involves IL12 and IFN‐G, which is strongly suppressed by IL10. This action helps to shift the cellular immune response from anti‐inflammatory process of Th1 to Th2 via adrenergic agonists as a result of stress. Moreover, the immunosuppressive effect is specific to the inflammatory cellular immune system. A shift from Th1 to Th2 cellular is strongly enhanced through the suppression of IL12, which is a major Th1 agonist within humoral immunity. However, the shift occurs proportionately rather than quantitatively. These effects above are observed over both the short and long term in PTSD. This decreased reaction of the immune system is also observed due to senescence with the chronic down‐regulation of cortisol receptors sites. The down‐regulation of cortisol receptors may reduce the capacity of lymphocytes to respond to anti‐inflammatory signals and allow other cytokine‐mediated processes to dominate in patients with PTSD.

## 2 IMMUNOSUPPRESSION AND SUSCEPTIBILITY TO COVID‐19

Many clinical observations have shown that elderly patients, those with an underlying chronic disease and treated with immunosuppressants, or patients otherwise in an immunosuppressed state could suffer from a decreased immune response and greater susceptibility to life‐threatening virus infections. Such infections show rapid national and international spread, such as in the case of SARS‐CoV‐2, which is currently posing a global health emergency.^5^, ^6^ The results of studies are particularly important for individuals who might be at a higher risk of developing complications that are associated with respiratory virus infections, such as the elderly, for whom the increased susceptibility to pathogens is a serious public health problem. Influenza and pneumonia are the fifth leading cause of mortality in individuals aged 50 or older who might have lower immunity.

Another clue has been showed that pregnancy is an independent risk factor to develop severe virus pneumonia under immune tolerance. The pregnancy bias toward Th2 system dominance which left pregnant woman vulnerable to viral infections might bring a challenge for the prevention of SARS‐CoV‐2 infection.^7^

**FIGURE 1:**
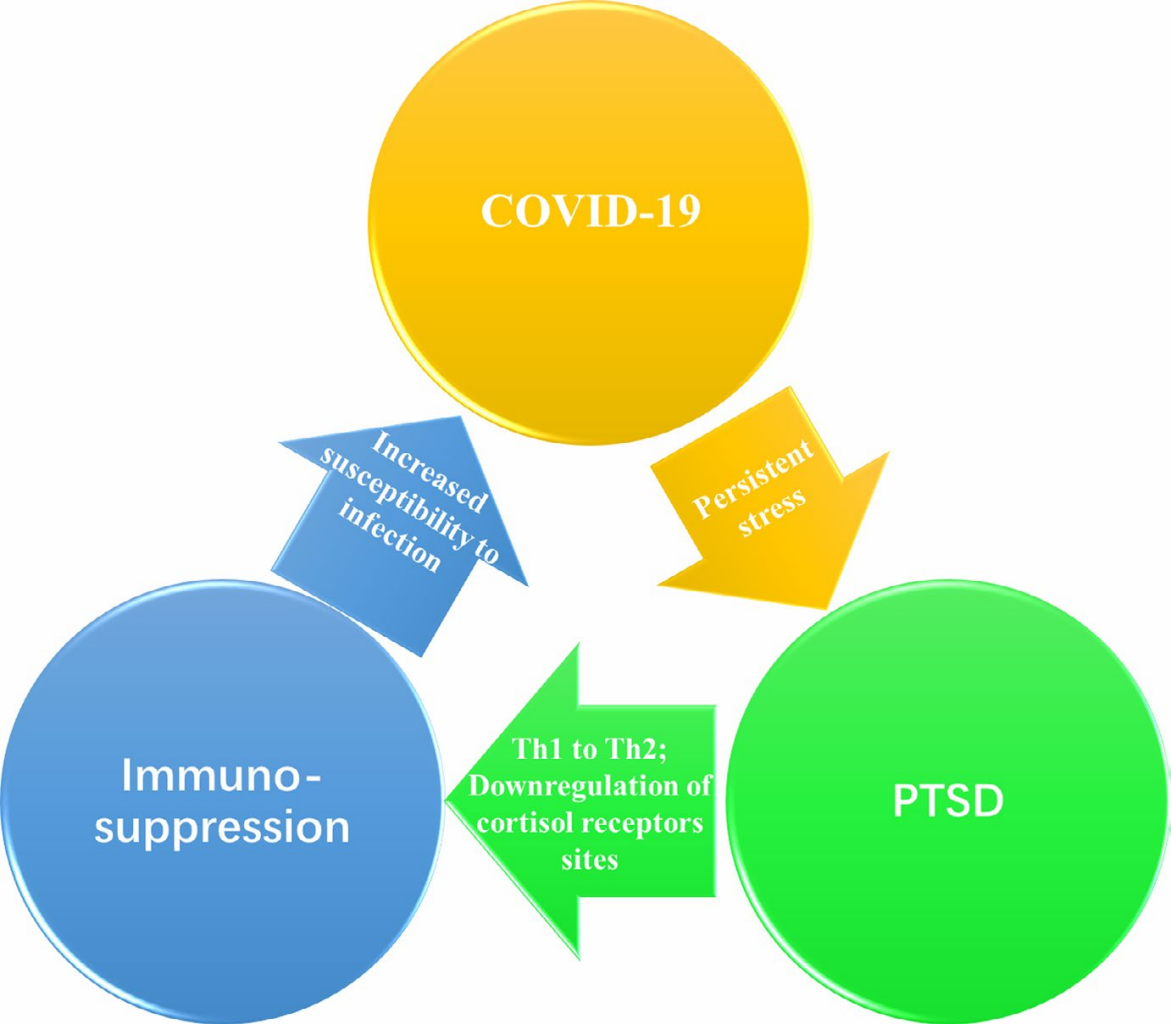
A vicious circle involving immunosuppression between COVID‐19 and PTSD

In addition, it has been showed that immunosuppression (secondary to disease or treatment) is by far the most identified risk factor to develop severe viral pneumonia by different respiratory virus families.^8^ Green ML has demonstrated that the profound and prolonged immunosuppression experienced by the patients undergoing hematopoietic cell transplantation and intensive chemotherapy for hematologic malignancy resulted in high rates of viral pneumonia that far surpassed the incidence in the general population.^9^ What is more, the rates of progression to pneumonia increase even depending on the risk factors of patients relating to the degree of immunosuppression (ie, lymphopenia, early post‐transplant, and use of immunosuppressive agents). SARS‐CoV‐2 causes COVID‐19 around the world accompaing with multiple psychological problems, such as PTSD in particular, in infected and healthy individuals. Subsequently, a vicious circle involving immunosuppression between COVID‐19 and PTSD may be engaged (Figure 1).

## 3 POSSIBLE STRATEGIES

During such a stressful period, psychological services and crisis interventions are needed at an early stage in almost all groups to reduce PTSD and in order to alleviate the current acute stress responses of individuals and patients and reduce the incidence of PTSD to prevent immunosuppression, thus breaking the vicious circle. Evidence‐based medicine is of great importance to conduct population‐based psychiatric surveys on the symptomatology of PTSD.^10^ Furthermore, neuroimaging can provide a heuristic framework for bridging gaps between thalamocortical neurocircuitry and depressive symptoms in PTSD.^11^, ^12^ Then, we can take appropriate psychological crisis intervention efficiently following the experience of China.^13^

## CONFLICT OF INTEREST

The authors have no conflicts of interest.

## Funding information

The work was supported by the National Key Research and Development Program of China (2016YFC1307100), the National Natural Science Foundation of China (81771465, 81930033), the Innovative Research Team of High‐level Local Universities in Shanghai, and the Key Program of Nantong University Clinical Medicine Special Project (2019JZ021).
